# Diffusion of cumulative advantage? How wealth and health trajectories co-evolve across the life course among older adults

**DOI:** 10.1093/geronb/gbag115

**Published:** 2026-06-27

**Authors:** Anastasia Lam, Philipp M Lersch

**Affiliations:** Faculty of Humanities and Social Sciences, Institute of Social Sciences, Humboldt-Universität zu Berlin, Berlin, Germany; Einstein Center Population Diversity, Berlin, Germany; Faculty of Humanities and Social Sciences, Institute of Social Sciences, Humboldt-Universität zu Berlin, Berlin, Germany; DIW Berlin, Socio-Economic Panel, Berlin, Germany

**Keywords:** Cumulative inequality theory, Growth curve modeling, Life domains, Chronic disease, Socioeconomic status

## Abstract

**Objectives:**

Cumulative advantage is a central concept in life course research. Prior research has primarily focused on one outcome domain, overlooking the possibility that cumulative advantage could extend into other domains—what we refer to as diffusion of cumulative advantage. We build on and extend cumulative inequality theory to conceptualize diffusion and apply it to wealth and health, two domains that are often considered prime examples of cumulative advantage, deriving novel expectations.

**Methods:**

To test our hypotheses, we draw on high-quality panel data from the Survey of Health, Ageing and Retirement in Europe (*N* = 45,221 individuals). Wealth is defined as liquid wealth, and health is defined as a count of 11 chronic conditions. We employ mixed-effects multivariate growth curve models to examine how wealth and health co-evolve over the life course among older adults by assessing covariances between the outcomes. We also stratify models by gender.

**Results:**

We found that while wealth generally increases with age, health declines. There was evidence for cumulative advantage within both wealth and health domains, and evidence for a diffusion of advantage from wealth to health and from health to wealth. Cumulative advantage in wealth and health and the diffusion of change across domains were stronger for women than for men.

**Discussion:**

The reinforcing relationships between wealth and health show how advantages can accumulate and diffuse across domains over the life course. These findings highlight the importance of understanding how multidimensional (dis)advantage unfolds over individuals’ lives, leading to the consolidation of inequalities over time.

Does cumulative advantage, that is, advantage begetting more advantage, diffuse across domains over the life course? For instance, do already wealthy individuals accumulate more wealth and, at the same time, experience more advantageous health changes over time? Answering such questions is fundamental for research on the life course and social inequality, as it helps understand how multidimensional advantage unfolds over individuals’ lives.

Theoretically, the life course perspective emphasizes multidimensionality, where different life course dimensions, such as work and family, are interwoven (Bernardi et al., 2019; Mayer, 2004). Building on this premise, cumulative inequality theory proposes that advantages gained in one domain can extend to others (Ferraro et al., 2009), reflecting the capacity of versatile resources, such as wealth, to function across domains. Despite its theoretical appeal, however, the notion of diffusion across domains remains conceptually underspecified, leaving open questions about how advantages translate from one domain to another.

Furthermore, expectations about the diffusion of advantage are rarely subjected to empirical testing. Research on cumulative advantage and inequality across the life course is mainly focused on one outcome domain and its subdomains, for example, health (Willson et al., 2007) and chronic diseases and functional limitations (Leopold, 2018). When multiple domains of advantage are studied, the focus is usually on how one (often categorical) domain of advantage, such as education, moderates cumulative advantage in another domain, such as income (Cheng, 2014). Such moderation is conceptually distinct from the diffusion of advantage across domains because of its unidirectional, single-outcome focus.

Therefore, we address the following research question: Do cumulative advantage and diffusion of cumulative advantage operate within and across wealth and health domains? In answering this question, we make three contributions. First, we build on cumulative inequality theory to advance the conceptualization of diffusion of advantage. In brief, we distinguish between diffusion of level (i.e., the level in one domain affects change in another domain) and diffusion of change (i.e., change in one domain affects change in another domain). Second, we demonstrate how multivariate growth curve models can be used to detect diffusion of advantage. Third, using wealth and health as the two outcome domains of interest, we are the first to empirically test for diffusion of advantage using high-quality data from the Survey of Health, Ageing, and Retirement in Europe (SHARE). Notably, the empirical strategy enables the study of the co-evolution of wealth and health, but it is unable to establish the causal direction of this influence.

The gradual outcomes of wealth and health are well-suited to studying cumulative advantage and the diffusion of cumulative advantage. First, both outcomes have been argued to be prime examples of cumulative advantage processes (DiPrete & Eirich, 2006; Willson et al., 2007). Second, it has also been argued that both outcomes are interdependent (Lynch & Brown, 2011). Third, wealth and health are key resources for successful aging that accumulate over time and are shaped by life course events (Oris et al., 2021). Thus, studying the reinforcing relationship between wealth and health highlights how advantages can accumulate and diffuse over the life course, leading to the consolidation of inequalities over time (Dannefer, 2003).

## Background

### A cumulative inequality framework for the diffusion of advantage

Accumulation is a concept central to studies on the life course as it describes how experiences, resources, or deficits build up over time. Based on this, Ferraro et al. (2009) introduced *cumulative inequality theory*, which describes the accumulation of advantages and disadvantages over the life course. This approach builds on earlier cumulative (dis)advantage theory (Dannefer, 2003) but is better suited for our purpose because it explicitly addresses cross-domain diffusion, considers subjective perceptions of change, clearly separates advantage from disadvantage, and provides a testable, axiomatized theory. Cumulative inequality theory was also proposed as a core framework for the field of gerontology to help understand how cumulative processes contribute to the development of inequalities within the context of population aging (Ferraro & Morton, 2018).

Cumulative inequality theory has five central axioms, of which we will focus on advancing numbers two and four: disadvantage leads to risk exposure, while advantage leads to opportunity exposure (axiom two), and the subjective perception of individuals’ trajectories impacts other trajectories (axiom four). Additionally, for ease of exposition, we focus on advantage in the development of our framework, which should not be considered to be the inverse of disadvantage (see Proposition 2a in Ferraro et al., 2009).

According to the second axiom, which is our primary focus, advantage accumulates for individuals because the current advantage opens opportunities to accrue further advantage. The simple example of compound interest demonstrates that greater wealth may open investment opportunities that yield higher returns, leading to quicker accumulation. Thereby, advantage begets more advantage. We define cumulative advantage as *level_w_* (in domain *w*) in time *t*_0_ having a (direct or indirect) positive effect on the change in *level_w_* until time *t*_1_ (following the strict form of cumulative advantage laid out in DiPrete & Eirich, 2006). In other words, the level affects growth rates in an outcome. This process is believed to occur within specific life domains (e.g., wealth begets more wealth) and may lead to different positions of advantage across domains. Notably, among older adults, cumulative advantage in outcomes such as wealth or health can result in a more gradual decline in wealth/health over time, rather than an improvement.

Advantage in one domain may also diffuse across domains (Proposition 2b in Ferraro et al., 2009). Opportunities provided through an advantage in one domain may extend to other domains. For instance, healthy individuals may be more physically active, slowing their health decline and simultaneously enabling them to work longer and accumulate more wealth. We draw inspiration from the concepts of “stress proliferation” (Pearlin et al., 2005, p. 209), spillover across the life course (George, 2003), and the life course cube (Bernardi et al., 2019) to describe the potential of cross-domain diffusion.

Within the original formulation of cumulative inequality theory, the concept of diffusion remains vague. We aim to extend the theory by clarifying the concept in the current study. We consider diffusion as a cumulative advantage process (i.e., advantage begets advantage) operating across domains. We propose that this can occur in two ways: diffusion of level and diffusion of change.

First, diffusion of cumulative advantage occurs when *level_w_* in *t*_0_ has a (direct or indirect) effect on the change in *level_h_* (in domain *h*) between *t*_0_ and *t*_1._ We label this *diffusion of level*. Diffusion of level is a dynamic process by which the level in one domain at a point in time unlocks opportunities for growth in another domain over time, distinct from mere co-occurrence of levels across domains at any point in time. Such a process may occur independently of whether a domain-specific cumulative advantage operates in domains *w* and *h*. In other words, those who have more in one domain gain more in another domain. For instance, the wealthy may purchase superior healthcare to improve their health more quickly (or halt health decline) than the poor. Similarly, the healthy may be more likely to work and save from surplus income and, at the same time, have less health expenditure, which would also increase their saving potential.

Second, the change in *level_w_* between *t*_0_ and *t*_1_ may positively affect the change in *level_h_* between both time points, which we denote as *diffusion of change*. This implies a dynamic coupling of change over time, independent of levels. In other words, those who gain more in one domain also gain more in another. For instance, those accumulating wealth more quickly may subjectively perceive this as a more successful trajectory (e.g., a sense of control, reduced anxiety about the future), which, in turn, improves their mental health and well-being more than for those on less steep wealth trajectories. This argument builds on the fourth axiom of cumulative inequality theory. Empirically, such expectations are also supported by findings for income and health, where men have been found to gain additional health advantages from being upwardly mobile in income (Frech & Damaske, 2019) and income changes have been found to be associated with health changes independent of income levels (Reed et al., 2025). Similarly, literature on intergenerational educational mobility and changes in cognitive functioning reports links across domains (Shi et al., 2024).

More generally, diffusion of level captures how structural position in one domain shapes mobility in another. Diffusion of change captures how mobility trajectories themselves become synchronized, where advantage accumulation in one domain propels advantage accumulation in another, independent of absolute position. The former is about what resources individuals have to convert. The latter is about how the experience of change itself transforms individuals—how being on a trajectory reshapes dispositions and capacities, independent of destination (Bourdieu, 1984, p. 110, 1986).

What we do not consider as diffusion of advantage is a positive correlation in levels across domains *w* and *h* at *t*_0_. A positive correlation across domains can capture prior life course processes, but does not indicate cross-domain diffusion. Instead, it would indicate a third (potentially unobserved) factor affecting both domains. In this case, family background and intergenerational reproduction of advantage would be obvious candidates (Proposition 1c in Ferraro et al., 2009). We will refer to the correlation in levels across domains as *clustering of advantage* rather than diffusion.

### The reinforcing relationship between wealth and health: Empirical evidence

Socioeconomic status is one of the most important determinants of health, with higher socioeconomic status being associated with better health (Mackenbach et al., 2008; Marmot & Bell, 2012). While income may represent only a snapshot of an individual’s current financial situation, wealth is a stock that can be built up over time, thereby providing more security in old age when income is no longer present. Therefore, it may play a more important role for old-age health than other measures of socioeconomic status, even across a wide range of health measures (Pollack et al., 2007). The relationship between wealth and health can also be bidirectional, with health playing a crucial role in labor force participation. Individuals with poorer health are more likely to exit employment early (Gurgel do Amaral et al., 2022; Van Zon et al., 2020), thereby having less time to accumulate wealth. However, some individuals may continue working into older age despite having poor health due to financial need (Di Gessa et al., 2018). Previous research also documents the direct negative consequences of health shocks and health selection for wealth in the U.S. (Haas, 2006; Lee & Kim, 2003; Smith, 1999; Wilkinson et al., 2019).

These interdependencies between wealth and health may be further complicated by gender. For instance, women have longer life expectancy, but they also spend more of their lives in poor health compared to men (di Lego et al., 2019; Oksuzyan et al., 2010; Oksuzyan et al., 2010). Conversely, while women are generally healthier, men are more likely to be wealthier, which is likely driven by traditional gender norms (Lersch et al., 2017; Meriküll et al., 2021; Sierminska et al., 2010). Although gender differences in wealth and health are well-studied separately, it is less clear how gender patterns may differ when considering combined wealth and health domains.

### The current study

We aim to answer the research question: Do cumulative advantage and diffusion of advantage operate within and across wealth and health domains? Accordingly, we test the following hypotheses. Cumulative advantage occurs in the wealth domain, that is, those with more wealth experience more positive change (increase or less decline) in their wealth over time (*H1a: cumulative advantage in wealth*). Cumulative advantage also occurs in the health domain, where individuals who are healthier tend to experience more positive changes (less decline) in their health over time (*H1b: cumulative advantage in health*). Wealth and health levels are correlated (*H2: clustering of advantage*). Cumulative advantage diffuses from wealth to health; that is, those wealthier see more positive change (less decline) in their health (*H3a: diffusion from wealth*). Cumulative advantage diffuses from health to wealth, that is, those healthier see more positive change (less decline) in their wealth (*H3b: diffusion from health*). Those with more positive change (less decline) in wealth over time will experience more positive change (less decline) in health and vice versa (*H3c: diffusion of change*). Given that prior evidence suggests substantial gender disparities in health and wealth, we also examine potential heterogeneity by gender; however, we treat this as an additional exploratory analysis rather than trying to test specific hypotheses.

## Method

To test our hypotheses, we draw on data from SHARE, a European longitudinal survey that gathers information on health, socioeconomic status, and social and family characteristics for adults aged 50 and older and their spouses of any age (Bergmann et al., 2019; Börsch-Supan et al., 2013). We use a harmonized dataset provided by the Gateway to Global Aging Data (Wilkens et al., 2024). The initial sample consisted of 85,733 individuals (*n* = 168,383 observations) aged 40–64 years from 29 countries across seven waves (1–2, 4–6, 8–9; years 2004–2022). Observations were excluded if they were missing marital status (*n* = 74 individuals; 137 observations), urban/rural residence (*n* = 607 individuals; 2,006 observations), birth country (*n* = 403 individuals; 660 observations), work status (*n* = 338 individuals; 846 observations), and co-residence with children (*n* = 339 individuals; 1,318 observations). We also excluded any individuals who only had one observation (*n* = 38,651), which subsequently dropped Ireland from the sample, and any households that had more than one couple to more clearly define couple-level wealth (*n* = 100 individuals; 520 observations), resulting in a final analytic sample of 45,221 individuals (*n* = 124,245 observations) from 28 countries.

### Measures

*Wealth* is time-varying and defined as couple-equivalized liquid wealth, which is the sum of savings, checking, bond, and stock wealth minus debt (excluding mortgages). We focus on liquid wealth (excluding self-used housing), which can be more readily used to improve health. Liquifying housing wealth is rare among older adults in Europe (Chiuri & Jappelli, 2010; Haurin & Moulton, 2017; Suari‐Andreu et al., 2019). Couple-level wealth was chosen instead of household-level wealth to avoid including the wealth of adult children living in the household. It was divided by the number of adults in the couple who are present in the household to get the equivalized amount under the assumption of equal sharing within the household. We use the imputed wealth measures provided by SHARE due to the high levels of non-response for monetary variables in the surveys (De Luca et al., 2015). Wealth was adjusted using exchange rates and purchasing power parity provided by SHARE, winsorized at the 0.01 and 99.9 percentiles, and is expressed in 1,000 Euros. We also examine an alternative operationalization using an inverse hyperbolic sine (IHS) transformation (Friedline et al., 2015), which is presented in Supplementary material Section 2. Additionally, we report a robustness check using total wealth (including self-used housing). Results of robustness checks are discussed below.

*Health* is time-varying and defined as a count of the following 11 chronic conditions: hypertension, diabetes, cancer, lung disease, heart problems, stroke, psychiatric disorders, arthritis, high cholesterol, Parkinson’s Disease, and Alzheimer’s Disease or Dementia. These were measured by asking participants whether a doctor had ever told them they had or currently have these conditions. All conditions were asked about in each wave, except for psychiatric disorders and Alzheimer’s Disease/Dementia, which were asked about starting in wave 2. The count scale is flipped to align with the wealth outcome, so that higher numbers indicate fewer conditions (i.e., better health). We conduct an additional robustness check using a frailty index to test the sensitivity of our hypotheses to an alternative health measure (see Supplementary Table 3.2 for further details).

The underlying time variable for our growth curve models (see below) is *age* in years, centered at the sample mean (57.4 years). We conduct an additional robustness check centered at age 50 (the age at which the main participants can enter the survey). To model nonlinear growth, we also include a quadratic term for *age.*

Control variables include *woman* (yes = 1, no = 0), *married or partnered* (yes = 1, no = 0), *no children* (yes = 1, no = 0), *co-resident with children* (yes = 1, no = 0), *rural* (rural = 1, urban = 0), *born in survey country* (yes = 0, no = 1), *work status* (working = 1, not working = 0), *income* (household equivalized), and categorical variables for *education* (less than upper secondary, upper secondary/vocational, tertiary), and *country.* Gender, birth country, and education are time-constant; all other variables are time-varying. We also add *wave* fixed effects. Descriptive statistics are presented in Supplementary Table 1.1.

### Statistical analysis

Multivariate growth curve models (Grimm et al., 2017) are used to assess the trajectories of wealth and health over age, examine covariances between wealth and health, and investigate how these parameters differ by gender. We use covariances in these models rather than simple bivariate correlations to capture diffusion because they allow us to quantify dynamic processes over time and separate the types of diffusion we are interested in. In contrast, bivariate correlations would conflate diffusion of levels, diffusion of change, and clustering of advantage.

In our models, repeated measures are included at level 1, and individuals are included at level 2. The control variables are included as fixed effects, and linear age is included as a random effect for both outcomes at level 2. Quadratic age is included as a random effect for a robustness check, but not in the main specification, to reduce complexity (Supplementary Table 3.5). The equation for the multivariate model can be written as


$${\mathrm{wealth}}_{it}=b_{0}+{b_{1}{\mathrm{age}}_{it}+b_{2}{\mathrm{age}}_{it}^{2}+Z_{it}^{\;}\delta_{\;}+u}_{0i}+u_{1i}{\mathrm{age}}_{it}+\varepsilon_{1it}^{\;},$$

$${\mathrm{health}}_{it}=b_{3}+b_{4}{\mathrm{age}}_{it}+b_{5}{\mathrm{age}}_{it}^{2}+Z_{it}^{\;}\gamma_{\;}+{u_{2i}+u}_{3i}{\mathrm{age}}_{it}+\varepsilon_{2it}^{\;},$$

[u0iu1iu2iu3i]∼MVN(0,Ωu):Ωu= [σu02   σu1,u0σu12  σu2,u0σu2,u1σu22 σu3,u0σu3,u1σu3,u2σu32]

where *wealth_it_* and *health_it_* are the outcomes for individual *i* at observation time *t*. *b*_0_ and *b*_3_ are the mean intercepts for wealth and health, respectively, and *b*_1_, *b*_2_, *b*_4_, and *b*_5_ represent the fixed effects of age on each outcome. ***Z***_*it*_ is a vector of additional control variables with regression coefficients $\delta_{\;}$ and γ. Between-individual variation is modeled by random effects for intercepts and age for wealth (*u*_0__*i*_, *u*_1__*i*_) and health (*u*_2__*i*_, *u*_3__*i*_). $\varepsilon_{1it}^{\;}$ and $\varepsilon_{2it}^{\;}$ are the residual error terms that capture within-individual variation across observations. The four random terms (*u*_0__*i*_, *u*_1__*i*_, *u*_2__*i*_, *u*_3__*i*_) are assumed to follow a joint multivariate normal distribution with mean zero and a variance-covariance matrix Ω_u_.

We can interpret relevant covariances as evidence for cumulative advantage under the assumption of no unobserved confounding. In our model, the relationship between wealth and health is captured through random effects; we do not incorporate wealth directly into the health equation or vice versa, acknowledging their endogeneity. SHARE multiply imputes the wealth data using a Fully Conditional Specification (De Luca et al., 2015). We pool the five implicates following Rubin’s rules to obtain a single point estimate. Variances are log-transformed before pooling, pooled on the log scale, then back-transformed (Toutenburg & Rubin, 1990).

Table 1 links each hypothesis with the parameter used to test it in the model. To test our hypotheses, we first build a simple model that does not allow any covariance between the intercepts and slopes (Model 1). We then allow additional parameters to covary in a stepwise manner, building increasingly complex models: intercept-slope in wealth (Model 2), intercept-slope in health (Model 3), intercepts across outcomes (Model 4), slopes across outcomes (Model 5), intercept wealth and slope health (Model 6), and intercept health and slope wealth (Model 7). For each step, we evaluate whether the model fit, as measured by the Bayesian information criterion (BIC) which weighs fit against model complexity, has increased. A difference in BIC of at least 2 is considered evidence for a better fitting model (a difference of at least 6 is considered strong evidence, and at least 10 very strong) (Raftery, 1995). For reference, we also report model fit based on the Akaike information criterion (AIC) and model deviance.

**Table 1:** Overview of hypotheses and empirical tests.

Hypothesis	Description	Parameter in the multivariate growth curve model
**H1a**	Cumulative advantage in wealth	$\sigma_{u1,u0}$ (Covariance intercept-slope for wealth)
**H1b**	Cumulative advantage in health	$\sigma_{u3,u2}$ (Covariance intercept-slope for health)
**H2**	Clustering of advantage	$\sigma_{u2,u0}$ (Covariance in intercepts)
**H3a**	Diffusion of advantage from wealth to health	$\sigma_{u3,u0}$ (Covariance wealth intercept—health slope)
**H3b**	Diffusion of advantage from health to wealth	$\sigma_{u2,u1}$ (Covariance health intercept—wealth slope)
**H3c**	Diffusion of change	$\sigma_{u3,u1}$ (Covariance wealth slope—health slope)

After estimating the pooled models, we also estimate the models separately by gender to recover the gender-specific variance-covariance matrix (but do not directly test for gender interactions). We report additional robustness checks below. We use R version 4.4.1 (R Core Team, 2024) for data preparation and Stata 17.0 (StataCorp, 2021), the runmlwin program (Leckie & Charlton, 2012), and MLwiN 3.13 (Charlton et al., 2024) to run the multivariate growth models.

## Results

### Wealth and health trajectories and diffusion of advantage

**Figure 1:**
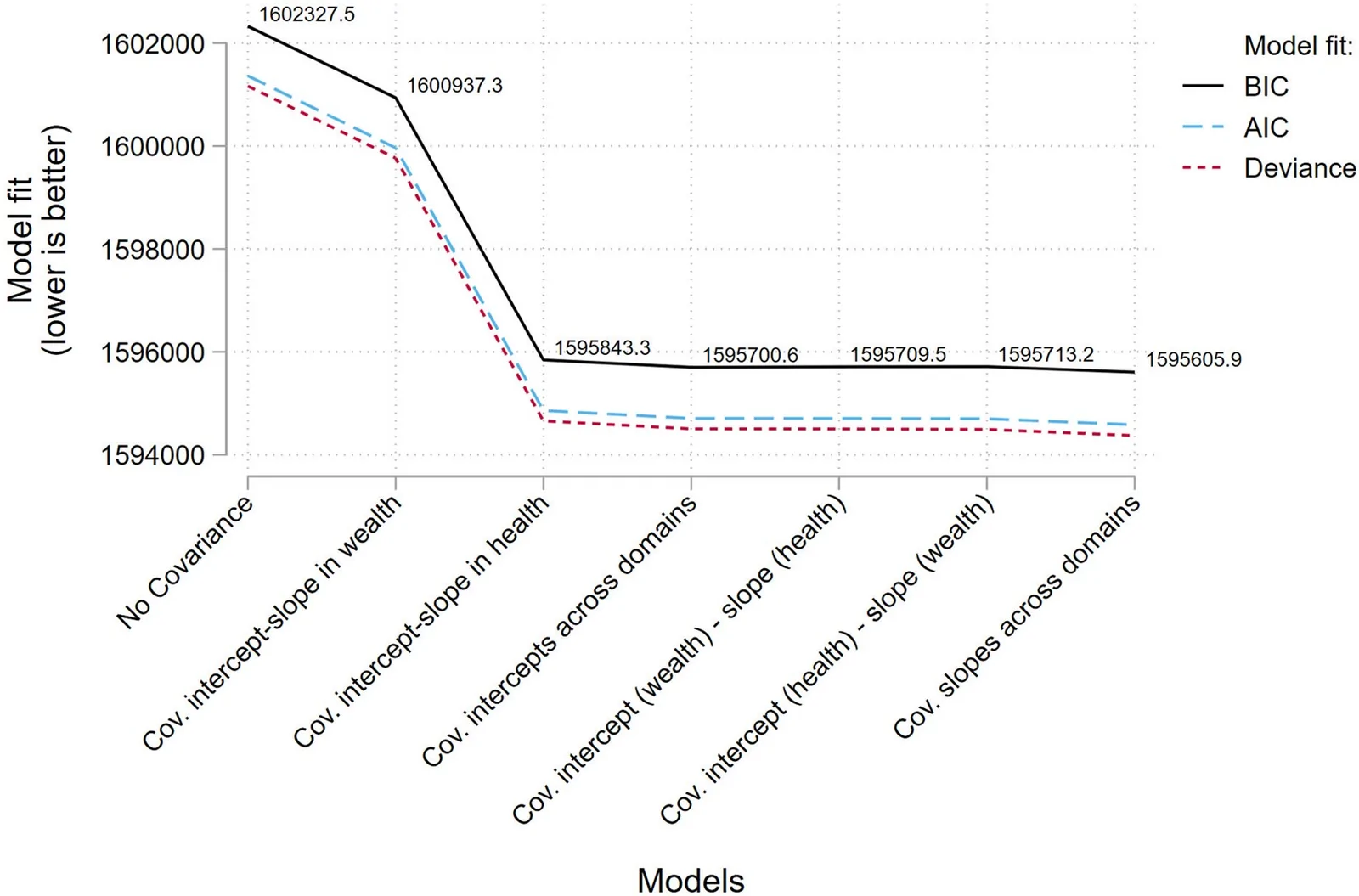
Model fit statistics for multivariate growth curve models with increasing intercept-slope covariance parameters. AIC = Akaike information criterion; BIC = Bayesian information criterion; Cov. = Covariance.

To test our hypotheses, we begin with a growth curve model in which all covariances are constrained to be zero. Allowing the covariance between intercepts and slopes for the wealth outcome to be freely estimated very strongly improves model fit based on BIC, as predicted in H1a (Figure 1). However, at this stage, we have not yet evaluated the direction of association indicated by the covariance. Also allowing most other covariances—between intercepts and slopes for the outcome health (H1b), between intercepts across outcomes (H2), and between slopes across outcomes (H3c)—to be freely estimated strongly improves model fit. The only exceptions are the covariance between intercepts in wealth and slopes in health (H3a) and between intercepts in health and slopes in wealth (H3b), which do not increase model fit. Thus, based on the BIC, the best-fitting model is one that comprehensively accounts for cross-domain relationships in wealth and health, but not for diffusion from wealth or diffusion from health.

Table 2 presents the estimated fixed and random parameters for the best-fitting model. We find that, on average, the fixed parameters indicate that wealth increases by approximately EUR 15,200 in the first 10 years, with gains accelerating until year 18, combining the linear and squared terms for age. The mean wealth over the study period is EUR 16,830 (with a standard deviation of EUR 43,070). In contrast, the health estimate falls by 0.75 (on a scale of 0 to 11) over the first ten years. We find considerable variance around the average age slope for wealth, as indicated by the variance in random slopes.

**Table 2:** Results from pooled multivariate growth curve model of wealth and health, using data from the Survey of Health, Aging, and Retirement in Europe, 2004–2022.

Variables		Estimate	95% CI	Correlation
**Fixed-effects parameters**				
**Wealth**	Intercept	−2.42	−4.58, −0.26	
	Age	1.12	1.04, 1.20	
	Age^2^	0.04	0.03, 0.05	
**Health**	Intercept	10.12	10.06, 10.18	
	Age	−0.07	−0.07, −0.07	
	Age^2^	−0.00	−0.00, −0.00	
**Random-effects parameters**				
**Level 2: individual**				
**Variance**	Wealth intercept	645.30	628.98, 661.63	
	Wealth slope	5.86	5.31, 6.42	
	Health intercept	1.16	1.15, 1.18	
	Health slope	0.01	0.01, 0.01	
**Covariance**	Intercept-slope in wealth ($\sigma_{u1,u0})$	32.01	29.77, 34.26	0.52
	Intercept-slope in health ($\sigma_{u3,u2})$	0.05	0.05, 0.06	0.57
	Intercepts across domains ($\sigma_{u2,u0})$	2.82	2.45, 3.19	0.10
	Intercept wealth—slope health ($\sigma_{u3,u0})$	0.24	0.19, 0.28	0.10
	Intercept health—slope wealth ($\sigma_{u2,u1})$	0.09	0.02, 0.16	0.03
	Slopes across domains ($\sigma_{u3,u1})$	0.02	0.01, 0.03	0.08
**Level 1: observation**				
**Variance**	Wealth residuals	1062.11	1035.47, 1088.75	
	Health residuals	0.25	0.25, 0.26	
**Covariance**	Residuals across domains	−0.10	−0.24, 0.04	−0.01

*Note*. CI = confidence interval. The control variables woman, married or partnered, no children, co-residence with children, rural, born in survey country, working, household equivalized income, education, country, and wave are included in the model. Age is centered at 57.4 years.

Next, we consider the covariance parameters to test our expectations. The covariance between the intercept and slope in wealth is positive, with a correlation of 0.52. This indicates that individuals with higher wealth have a faster wealth growth rate, which supports hypothesis H1a (cumulative advantage in wealth). Similarly, there is a positive correlation (0.57) between intercept and slope in health, which provides evidence for hypothesis H1b (cumulative advantage in health). We also find evidence for clustering of advantage (H2), with a positive correlation (0.10) between intercepts across domains. There is a positive covariance between intercept wealth and slope health, with a correlation of 0.10, and a positive covariance between intercept health and slope wealth, with a positive but substantially small correlation of 0.03, supporting H3a (diffusion from wealth) and H3b (diffusion from health), respectively. The correlation for slopes across domains is positive (0.08), providing evidence for a bidirectional diffusion of change (H3c) between wealth and health.

### How wealth and health trajectories differ by gender

Table 3 shows the estimated fixed and random parameters by gender for the best-fitting model. We observe that, on average, women gain more wealth with each year of age than men (linear term: 1,290 EUR vs. 950 EUR), but men and women have the same amount of health decline with each year of age (linear term: −0.07). Again, there is considerable variance around the average age slopes for wealth. The covariance parameters for men and women exhibit largely similar patterns to those of the pooled model, with a couple of notable differences. Women have a higher correlation for intercept-slope in wealth (0.64 vs. 0.29, more cumulative advantage in wealth) and for intercept-slope in health than men (0.60 vs. 0.52, more cumulative advantage in health). The correlations in intercepts across domains and slopes across domains are also higher for women than men (0.12 vs. 0.08 and 0.11 vs. 0.02, respectively), suggesting stronger clustering of advantage and diffusion of change among women.

**Table 3:** Results from multivariate growth curve models of wealth and health, by gender, using data from the Survey of Health, Aging, and Retirement in Europe, 2004–2022.

Variables		Men			Women		
		Estimate	95% CI	Corr	Estimate	95% CI	Corr
**Fixed-effects parameters**							
**Wealth**	Intercept	−0.31	−4.14, 3.51		−0.86	−3.79, 2.07	
	Age	0.95	0.81, 1.08		1.29	1.19, 1.38	
	Age^2^	0.03	0.01, 0.05		0.05	0.03, 0.06	
**Health**	Intercept	10.00	9.90, 10.09		10.16	10.08, 10.23	
	Age	−0.07	−0.07, −0.07		−0.07	−0.08, −0.07	
	Age^2^	−0.00	−0.00, −0.00		−0.00	−0.00, −0.00	
**Random-effects parameters**							
**Level 2: individual**							
**Variance**	Wealth intercept	675.71	649.89, 701.53		623.26	601.94, 644.58	
	Wealth slope	7.05	5.95, 8.14		5.70	5.11, 6.30	
	Health intercept	1.09	1.07, 1.12		1.21	1.19, 1.23	
	Health slope	0.01	0.01, 0.01		0.01	0.01, 0.01	
**Covariance**	Intercept-slope in wealth ($\sigma_{u1,u0})$	19.96	14.56, 25.37	0.29	38.41	36.06, 40.75	0.64
	Intercept-slope in health ($\sigma_{u3,u2})$	0.05	0.05, 0.05	0.52	0.06	0.05, 0.06	0.60
	Intercepts across domains ($\sigma_{u2,u0})$	2.13	1.58, 2.67	0.08	3.28	2.77, 3.78	0.12
	Intercept wealth—slope health ($\sigma_{u3,u0})$	0.23	0.15, 0.31	0.09	0.25	0.19, 0.30	0.12
	Intercept health—slope wealth ($\sigma_{u2,u1})$	0.10	−0.01, 0.22	0.04	0.08	−0.01, 0.18	0.03
	Slopes across domains ($\sigma_{u3,u1})$	0.00	−0.01, 0.02	0.02	0.02	0.01, 0.03	0.11
**Level 1: observation**							
**Variance**	Wealth residuals	1005.30	976.27, 1034.34		1094.76	1060.50, 1129.02	
	Health residuals	0.26	0.25, 0.26		0.25	0.25, 0.25	
**Covariance**	Residuals across domains	−0.03	−0.25, 0.18	0.00	−0.13	−0.33, 0.07	−0.01

*Note.* CI = confidence interval; Corr = correlation. The control variables woman, married or partnered, no children, co-residence with children, rural, born in survey country, working, household equivalized income, education, country, and wave are included in the model. Age is centered at 57.4 years.

### Robustness checks

Our results do not seem to be driven by individual countries, selective interview responses, or our chosen health measure. Estimates are robust when models were run excluding one country at a time (Supplementary Figure 2) and when univariate wealth and health models included sampling weights (Supplementary Table 3.1). Additionally, when we used a frailty index, the results were highly comparable to the main analysis (Supplementary Table 3.2). However, when we exclude countries from Central and Eastern Europe (which have more limited data availability), we see a larger correlation for intercept-slope in wealth (Supplementary Table 3.3).

When analyzing total wealth in place of liquid wealth, the largest differences compared to the main analysis are that the correlations for intercept-slope in wealth and the correlation between slopes across domains are weaker (Supplementary Table 3.4). Total wealth includes housing wealth, for which cumulative advantage is less likely, as no compound interest effects operate. When including IHS-transformed wealth, which transforms wealth to a multiplicative scale, we find no evidence for cumulative advantage in the wealth domain or diffusion of advantage from health (Supplementary Table 2.1).

When we included quadratic age as a random effect, evidence for cumulative advantage and diffusion of change is weaker, but other correlations are comparable (Supplementary Table 3.5). When we centered age at age 50 instead of at the sample mean, there is no longer evidence for cumulative advantage in both wealth and health, or diffusion of advantage from health (Supplementary Table 3.6). We will discuss these implications further in the discussion section.

Our findings are also robust when one partner is randomly selected from each couple (Supplementary Table 3.7), when we control for occupation type (Supplementary Table 3.8), and when we control for health behaviors (Supplementary Table 3.9). Lastly, when we stratify by working status, most observed differences relative to the main model are in effect size, but not in direction or significance (Supplementary Table 3.10).

## Discussion

In this study, we introduced a conceptual framework for the cross-domain diffusion of cumulative advantage, which extended existing cumulative inequality theory to better understand the multidimensional nature of life course inequalities. Using multivariate growth curve models, we investigated whether cumulative advantage and diffusion of advantage processes operated within and across wealth and health domains, and whether these processes varied by gender among older adults in Europe.

We found that while wealth generally increases with age, health declines. There was evidence of cumulative advantage in the wealth and health domains, with wealthier (healthier) individuals more likely to experience favorable changes (less decline) in their wealth (health) by age. Theoretically, this implies that wealth and health provide more opportunities for those in advantaged positions. It is essential to note that we proposed a specific and arguably narrow definition of cumulative advantage and empirically tested it. Our results do not address the diverse array of definitions for cumulative advantage found in the literature.

Our study is the first that we are aware of to test the concept of diffusion of advantage in assessing the bidirectional relationship between two outcomes: wealth and health. We find evidence of the diffusion of advantage across domains, whereby wealthy (healthy) individuals experience more favorable changes in health (wealth) over time. Thereby, we add to our understanding of the reinforcing relationship between wealth and health as one example of how the multidimensionality of life courses is reflected in the production of inequalities. Observed fanning out in life course outcomes among individuals over time can be driven by opportunities created by various reserves across domains.

Our results were largely similar when stratified by gender, but women had stronger correlations for cumulative advantage in wealth and health, and for the diffusion of change across domains. Women’s stronger cumulative advantage could indicate women using their wealth to support their health, and thereby their better health supports their wealth accumulation through investing in preventive measures or lifestyle, reflecting their higher healthcare-seeking compared to men (Galdas et al., 2005; Höhn et al., 2020).

Beyond our contributions to the understanding of cumulative advantage and the diffusion of advantage processes, we also advance our knowledge of wealth and health disparities and their relationships. Conceptually and empirically, we separate level and change effects, which are often conflated in prior research, and find that, in our sample, the correlations between level and change dominate the correlations in change across domains. At the same time, the correlations in change are non-negligible and indicate that the experience of change in wealth relates to change in health. This may suggest that individuals’ subjective perceptions of their trajectories influence other trajectories, as proposed in cumulative inequality theory.

A notable finding of our study is the sensitivity of our results on cumulative advantage and diffusion of advantage to age centering and the operationalization of wealth. First, the sensitivity to age centering reinforced the importance of one of Elder et al.’s (2003) key life course principles, the principle of timing, where cumulative advantage and diffusion of advantage processes are highly sensitive to the period of the life course being studied. Second, the sensitivity to the operationalization of wealth suggests that the scale and specific composition of resources under study are consequential. For instance, based on our theory, we expected cumulative advantage to operate on the absolute rather than the multiplicative scale (see Supplementary Material Section 2), but expectations may differ in other research contexts. These points limit the generalizability of our results. At the same time, they have received limited attention in prior research and highlight an important avenue for further research to better understand the conditions under which cumulative advantage and its diffusion occur.

Further limitations of our study are noteworthy. First, our data begins at age 40, and we lack detailed information on childhood wealth or health, so we cannot observe the wealth-health relationships earlier in life. Second, health is measured using only a count of 11 chronic conditions, so we are likely underestimating the number of potential health conditions individuals may have. Third, our approach is not suitable for establishing the causal effect of an acute change in one variable on the other. We also cannot explicitly establish the causal primacy of one variable over the other, but we assume that diffusion from one domain precedes change in the other domain.

The flexibility of our conceptual framework and empirical approach allows it to be applied to other life course processes and outcomes. Building on this foundation, we foresee three main avenues for future research. First, we use wealth and health as outcomes in this study, but it is unclear whether and how diffusion of advantage processes may function for other outcomes. And, as argued above, we need to extend our understanding of the specific conditions under which cumulative advantage occurs. Second, we identified that diffusion of advantage is sensitive to the life course stage, but this raises additional questions about what might happen to the cross-domain diffusion if there is some “shock” or “turning point” at which diffusion of advantage no longer holds. Third, in this study, we only focus on the concept of advantage. However, it is also important to determine whether patterns remain similar when extending the approach to the diffusion of disadvantage and the divergence of (dis)advantage, whereby advantage in one domain leads to disadvantage in another, or vice versa. Together with our study, answering these additional questions is essential to understanding how multidimensional (dis)advantage unfolds over individuals’ lives.

## Supplementary Material

gbag115_Supplementary_Data

## Data Availability

This study was not preregistered. After registration with the SHARE Data Research Center, the required data files can be downloaded here: https://releases.sharedataportal.eu. Code to run the analyses are made available at https://doi.org/10.17605/OSF.IO/J6G7M.
