# Supplementary material for: Diffusion of cumulative advantage? How wealth and health trajectories co-evolve across the life course among older adults
Source: J Gerontol B Psychol Sci Soc Sci. 2026 Jun 27;81(9):gbag115. doi: 10.1093/geronb/gbag115 (PMC13446222; doi:10.1093/geronb/gbag115)
Supplement: Supplementary file 1 [file gbag115_supplementary_data.zip › JGSS-suppl-Lam-Lersch.pdf]

The Journals of Gerontology, Series B: Psychological Sciences and Social Sciences
Supplementary Material: Lam & Lersch. Diffusion of cumulative advantage? How wealth and health trajectories co-evolve across the life course among older adults.

Section 1: Descriptive statistics

Supplementary Table 1.1 presents the descriptive statistics for our sample based on the entry wave of each individual overall and separately for women and men. We present additional information on the mean, median, 25th percentile, and 75th percentile for the wealth variable, pooled over the five imputations. Our sample consists of more women than men (58% vs. 42%). Approximately 75% of participants hold less than a tertiary education. Over 80% of participants are married or partnered, and about 10% of the sample do not have children. **Supplementary Table 1.2** shows the number of observations, waves, individuals, and couples per country, and **Supplementary Table 1.3** shows the number of individuals per country per wave. **Supplementary Figure 1** plots the age-specific median wealth and mean flipped chronic condition count (i.e., health). We see a continuous increase in wealth, especially after age 45. In contrast, we see a clear decline in health over time.

Supplementary Table 1.1. Descriptive statistics based on individuals' entry wave, for men, women, and overall

	Men (N=19,015)	Women (N=26,206)	Overall (N=45,221)
**Age (years)**			
Mean (SD)	55.5 (3.75)	54.5 (4.41)	54.9 (4.17)
**Education**			
Less than upper secondary	5,360 (28.2%)	8,363 (31.9%)	13,723 (30.3%)
Upper secondary and vocational	8,707 (45.8%)	11,287 (43.1%)	19,994 (44.2%)
Tertiary	4,948 (26.0%)	6,556 (25.0%)	11,504 (25.4%)
**Married or partnered**			
Yes	16,150 (84.9%)	21,161 (80.7%)	37,311 (82.5%)
No	2,865 (15.1%)	5,045 (19.3%)	7,910 (17.5%)
**No children**			
Yes	2,211 (11.6%)	2,112 (8.1%)	4,323 (9.6%)
No	16,804 (88.4%)	24,094 (91.9%)	40,898 (90.4%)
**Co-residence with children**			
Yes	8,595 (45.2%)	11,564 (44.1%)	20,159 (44.6%)
No	8,209 (43.2%)	12,530 (47.8%)	20,739 (45.9%)
Missing (no children)	2,211 (11.6%)	2,112 (8.1%)	4,323 (9.6%)
**Rural**			
Yes	6,524 (34.3%)	8,502 (32.4%)	15,026 (33.2%)
No (Urban)	12,491 (65.7%)	17,704 (67.6%)	30,195 (66.8%)
**Born in survey country**			
Yes	17,209 (90.5%)	23,485 (89.6%)	40,694 (90.0%)
No	1,806 (9.5%)	2,721 (10.4%)	4,527 (10.0%)
**Working**			
Yes	13,585 (71.4%)	15,430 (58.9%)	29,015 (64.2%)
No	5,430 (28.6%)	10,776 (41.1%)	16,206 (35.8%)
**Equivalized household income (in 1000 EUR)**			
Mean (SD)	287 (591)	278 (586)	282 (589)
Median [p25, p75]	130 [48.6, 305]	122 [45.9, 287]	125 [46.8, 291]
**Wealth (in 1000 EUR)***			
Mean (SD)	13.8 (40.3)	13.3 (39.2)	13.5 (39.7)
Median [p25, p75]	1.67 [0, 13.6]	1.54 [0, 12.7]	1.59 [0, 13.0]
**Disease count (flipped, 0-11)**			
Mean (SD)	10.1 (1.10)	10.1 (1.13)	10.1 (1.12)
Median [p25, p75]	10.0 [10.0, 11.0]	10.0 [10.0, 11.0]	10.0 [10.0, 11.0]

p25: 25th percentile, p75: 75th percentile, SD: standard deviation
* Pooled over five imputations

Supplementary Table 1.2. Number of observations, waves, individuals, and couples per country

Country	N observations	Avg. N waves	N individuals	N couples
Austria	6,734	3.1 (out of 7)	2,399	1,284
Belgium	12,313	3.4 (out of 7)	4,140	2,270
Bulgaria	408	2 (out of 2)	204	128
Croatia	2,117	2.4 (out of 3)	916	520
Cyprus	134	2 (out of 2)	67	36
Czechia	7,315	3.1 (out of 6)	2,563	1,502
Denmark	8,256	3.5 (out of 7)	2,691	1,577
Estonia	8,432	3.1 (out of 5)	3,033	1,829
Finland	412	2 (out of 2)	206	100
France	9,789	3.4 (out of 7)	3,296	1,935
Germany	8,824	3.1 (out of 7)	3,169	1,780
Greece	6,254	2.7 (out of 5)	2,501	1,504
Hungary	955	2.2 (out of 3)	440	232
Israel	3,365	2.9 (out of 6)	1,290	770
Italy	8,314	3.3 (out of 7)	2,822	1,840
Latvia	710	2 (out of 2)	355	206
Lithuania	874	2 (out of 2)	437	256
Luxembourg	1,751	2.7 (out of 4)	691	300
Malta	350	2 (out of 2)	175	110
Netherlands	5,636	2.9 (out of 6)	2,099	1,152
Poland	3,925	2.5 (out of 5)	1,664	996
Portugal	1,378	2.3 (out of 3)	615	372
Romania	790	2 (out of 2)	395	246
Slovakia	842	2 (out of 2)	421	304
Slovenia	5,544	3 (out of 5)	2,040	1,096
Spain	7,295	3.1 (out of 7)	2,617	1,657
Sweden	5,732	3 (out of 7)	2,130	1,022
Switzerland	5,796	3.5 (out of 7)	1,845	970

Supplementary Table 1.3. Number of individuals per country and wave

Country	Wave						
	1	2	4	5	6	8	9
Austria	516	512	1,874	1,774	1,117	449	492
Belgium	1,555	1,655	2,303	2,501	2,388	781	1,130
Bulgaria	-	-	-	-	-	204	204
Croatia	-	-	-	-	596	708	813
Cyprus	-	-	-	-	-	67	67
Czechia	-	572	1,939	2,111	1,732	576	385
Denmark	710	1,167	1,188	1,854	1,770	824	743
Estonia	-	-	2,279	2,165	1,960	972	1,056
Finland	-	-	-	-	-	206	206
France	1,152	1,308	2,280	1,960	1,571	871	647
Germany	787	981	619	2,197	2,054	1,204	982
Greece	1,317	1,537	-	-	1,628	963	809
Hungary	-	-	247	-	-	367	341
Israel	831	899	-	817	595	150	73
Italy	862	1,203	1,296	1,726	1,758	638	831
Latvia	-	-	-	-	-	355	355
Lithuania	-	-	-	-	-	437	437
Luxembourg	-	-	-	514	644	355	238
Malta	-	-	-	-	-	175	175
Netherlands	1,097	1,280	1,245	1,266	-	414	334
Poland	-	760	771	-	734	815	845
Portugal	-	-	590	-	602	-	186
Romania	-	-	-	-	-	395	395
Slovakia	-	-	-	-	-	421	421
Slovenia	-	-	1,021	1,218	1,433	949	923
Spain	665	866	1,336	2,050	1,771	415	192
Sweden	1,071	1,139	592	1,197	1,026	403	304
Switzerland	347	597	1,514	1,348	1,063	568	359

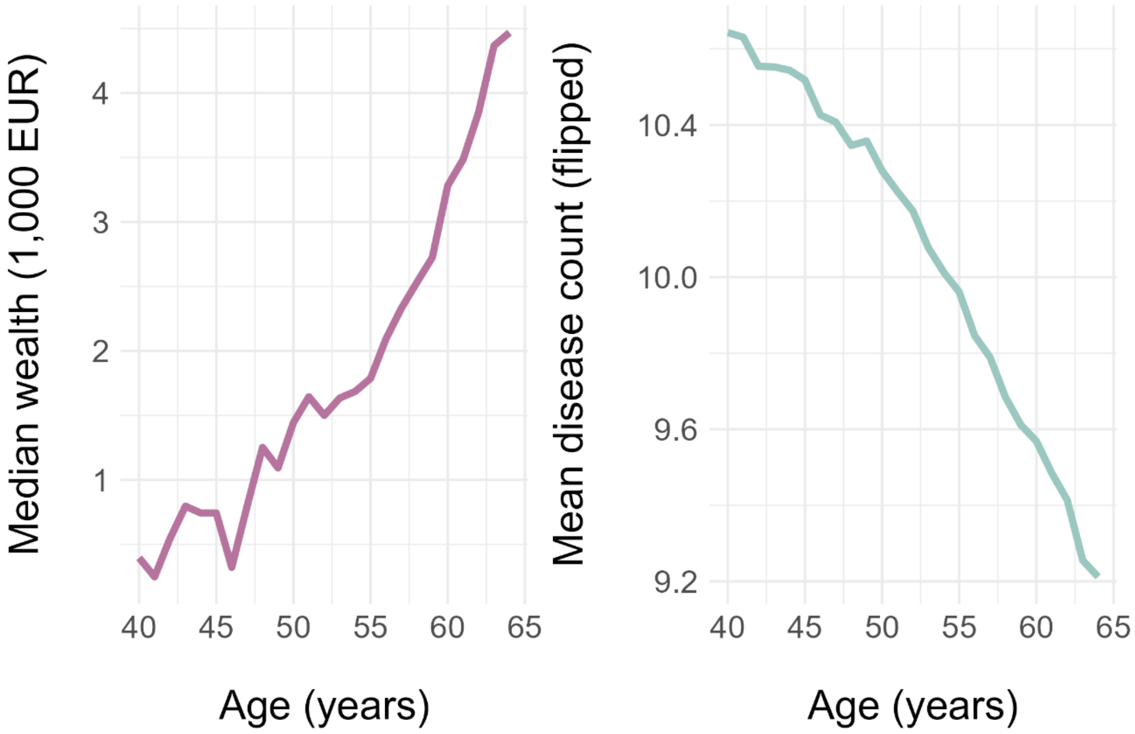

Supplementary Figure 1. Age-specific median wealth (per 1,000 EUR) and mean disease count (flipped, range: 0-11), using data from the Survey of Health, Aging, and Retirement in Europe, 2004-2022. Wealth is the average of five imputed values. Disease count is presented on a flipped scale, so higher values of indicate less disease.

Section 2: Alternative operationalization of wealth

Instead of raw wealth, wealth can be transformed to account for its skewed distribution. One popular approach is the inverse hyperbolic sine (IHS) transformation (Burbidge et al., 1988), which is recommended when studying wealth (Pence, 2006). This transformation has the following form:

$$x' = log(x + \sqrt{x^2 + 1}) = arcsinh(x)$$

This function is defined for zeros and negative values, is linear at the origin, and is symmetric about zero. Importantly, similar to a log-transformation, when using the IHS-transformation, multiplicative effects are estimated in regressions. We decided against using the IHS-transformation in the main analysis, because based on our theory, we would expect cumulative advantage to operate on the absolute scale rather than a multiplicative scale. In addition, to maintain comparability with the health domain, it is also important not to transform wealth.

Supplementary Table 2 shows results when using inverse hyperbolic transformed wealth. Using this alternative wealth transformation, we do not find evidence for cumulative advantage as the covariance between wealth intercept and wealth slope is negative. This is also the case for the covariance between health intercept and wealth slope, and slopes across domains.

Another common approach is the rank transformation, where observations are ordered by wealth and are assigned their percentile rank position in the annual distribution. The rank transformation provides a relative ranking of observations where vastly different absolute distances can be translated into similar relative percentile distances. Because of its simplicity, the rank transformation is attractive. However, the year-specific ranking of individuals may mask cumulative advantage processes if these processes lead to a fanning out of absolute levels but do not change relative positions within distributions.

Supplementary Table 2. Estimated coefficients, 95% confidence intervals, and correlations from pooled multivariate growth curve model of *IHS-transformed wealth* and health, using data from the Survey of Health, Aging, and Retirement in Europe, 2004-2022

**Fixed-effects parameters**				
		Estimate	*95% CI*	
Wealth	Intercept	2.71	2.36 - 3.05	
	Age	0.24	0.22 - 0.25	
	Age^2	0.00	-0.00 - 0.00	
Health	Intercept	10.13	10.07 - 10.19	
	Age	-0.07	-0.07 - -0.07	
	Age^2	-0.00	-0.00 - -0.00	
**Random-effects parameters**				
Level 2: individual		*Estimate*	*95% CI*	*Correlation*
Variance	Wealth intercept	14.83	14.43 - 15.22	
	Wealth slope	0.09	0.08 - 0.10	
	Health intercept	1.16	1.15 - 1.18	
	Health slope	0.01	0.01 - 0.01	
Covariance	Intercept-slope in wealth ($\sigma_{u1,u0}$)	-0.61	-0.65 - -0.57	-0.53
	Intercept-slope in health ($\sigma_{u3,u2}$)	0.05	0.05 - 0.06	0.57
	Intercepts across domains ($\sigma_{u2,u0}$)	0.64	0.58 - 0.70	0.15
	Intercept wealth – slope health ($\sigma_{u3,u0}$)	0.04	0.04 - 0.05	0.13
	Intercept health – slope wealth ($\sigma_{u2,u1}$)	-0.02	-0.03 - -0.01	-0.07
	Slopes across domains ($\sigma_{u3,u1}$)	-0.00	-0.00 - 0.00	-0.01
Level 1: observation		*Estimate*	*95% CI*	*Correlation*
Variance	Wealth residuals	30.30	29.91 - 30.69	
	Health residuals	0.25	0.25 - 0.26	
Covariance	Residuals across domains	0.01	-0.01 - 0.03	0.00

Note. The control variables woman, married or partnered, no children, co-residence with children, rural, born in survey country, working, household equivalized income, education, country, and wave are included in the model. Age is centered at 57.4 years.

References

Burbidge, J. B., Magee, L., & Robb, A. L. (1988). Alternative Transformations to Handle Extreme Values of the Dependent Variable. *Journal of the American Statistical Association*, *83*(401), 123–127. https://doi.org/10.2307/2288929

Pence, K. M. (2006). The Role of Wealth Transformations: An Application to Estimating the Effect of Tax Incentives on Saving. *The B.E. Journal of Economic Analysis & Policy*, *5*(1), 0000101515153806451430. https://doi.org/10.1515/1538-0645.1430

Section 3: Other Robustness checks

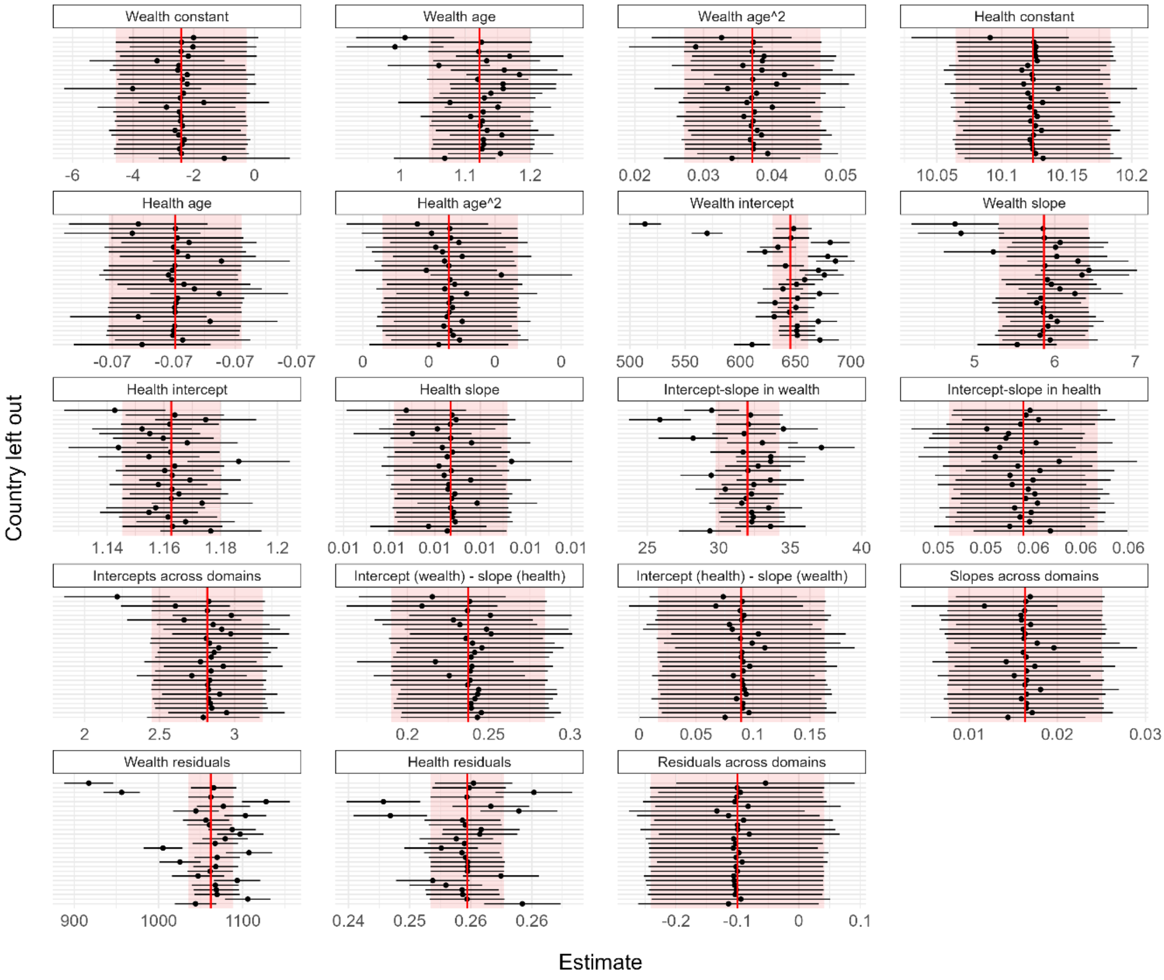

Supplementary Figure 2. Results from a leave-one-country-out analysis with estimated coefficients and 95% confidence intervals from pooled multivariate growth curve model (in black), using data from the Survey of Health, Aging, and Retirement in Europe, 2004-2022

Note. Each point represents the estimate when one country is excluded from the model. The lines represent the 95% confidence intervals. The red line and shaded area display the estimate and 95% confidence intervals from the main analysis, respectively.

Supplementary Table 3.1. Univariate wealth and health models with sampling weights, using data from the Survey of Health, Aging, and Retirement in Europe, 2004-2022

	Wealth		Health	
**Fixed-effects parameters**	*Estimate*	*95% CI*	*Estimate*	*95% CI*
Intercept	-2.47	-4.48 - -0.46	10.12	10.06 - 10.18
Age	1.15	1.07 - 1.23	-0.07	-0.07 - -0.07
Age^2	0.04	0.03 - 0.05	-0.00	-0.00 - -0.00
**Random-effects parameters**				
Level 2: Individual				
Variance: Intercept	658.87	620.77 - 696.97	1.15	1.13 - 1.18
Variance: Age	6.68	5.62 - 7.75	0.01	0.01 - 0.01
Covariance: Intercept-Age	24.14	19.31 - 28.96	0.05	0.05 - 0.05
Level 1: observation				
Variance: Intercept	1016.16	976.32 - 1055.99	0.25	0.24 - 0.25

Note. The control variables woman, married or partnered, no children, co-residence with children, rural, born in survey country, working, household equivalized income, education, country, and wave are included in the model. Age is centered at 57.4 years.

Supplementary Table 3.2. Estimated coefficients, 95% confidence intervals, and correlations from pooled multivariate growth curve model of liquid wealth and health measured using a *frailty index*, using data from the Survey of Health, Aging, and Retirement in Europe, 2004-2022

**Fixed-effects parameters**				
		Estimate	*95% CI*	
Wealth	Intercept	-2.17	-4.34 - -0.01	
	Age	1.12	1.04 - 1.20	
	Age^2	0.04	0.03 - 0.05	
Health	Intercept	86.64	86.21 - 87.07	
	Age	-0.31	-0.33 - -0.30	
	Age^2	0.00	0.00 - 0.01	
**Random-effects parameters**				
Level 2: individual		*Estimate*	*95% CI*	*Correlation*
Variance	Wealth intercept	647.33	631.05 - 663.61	
	Wealth slope	5.82	5.25 - 6.39	
	Health intercept	51.11	50.32 - 51.91	
	Health slope	0.23	0.22 - 0.25	
Covariance	Intercept-slope in wealth ($\sigma_{u1,u0}$)	31.98	29.74 - 34.21	0.52
	Intercept-slope in health ($\sigma_{u3,u2}$)	1.39	1.32 - 1.46	0.40
	Intercepts across domains ($\sigma_{u2,u0}$)	25.44	22.96 - 27.91	0.14
	Intercept wealth – slope health ($\sigma_{u3,u0}$)	0.88	0.57 - 1.19	0.07
	Intercept health – slope wealth ($\sigma_{u2,u1}$)	0.81	0.29 - 1.32	0.05
	Slopes across domains ($\sigma_{u3,u1}$)	0.09	0.03 - 0.15	0.08
Level 1: observation		*Estimate*	*95% CI*	*Correlation*
Variance	Wealth residuals	1063.02	1036.43 - 1089.61	
	Health residuals	18.14	17.94 - 18.35	
Covariance	Residuals across domains	-0.03	-1.22 - 1.16	0.00

Note. The control variables woman, married or partnered, no children, co-residence with children, rural, born in survey country, working, household equivalized income, education, country, and wave are included in the model. Age is centered at 57.4 years.

Health is time-varying and defined using a frailty index created using Theou et al.'s (2023) 10-step method. This consisted of identifying all health-related variables in the data, excluding variables with more than 5% missingness at any wave, recoding variables on a 0 to 1 scale, excluding variables in which deficits are either too rare or too common, assessing the variables' associations with age and correlations with other variables, counting the number of retained variables, then calculating the frailty index and using it in the analysis. Our frailty index consisted of a total of 58 variables, which include indicators of disability, functional limitations, chronic health conditions, medication use, cognitive impairment, depressive symptoms, and healthcare utilization. We flipped the index and multiplied it by 100 so that values closer to zero would indicate having more deficits and values closer to 100 would indicate better health. A 2-point decrease in our frailty index indicates a clinically meaningful decline in health (Theou et al., 2020).

Theou, O., Haviva, C., Wallace, L., Searle, S. D., & Rockwood, K. (2023). How to construct a frailty index from an existing dataset in 10 steps. *Age and Ageing, 52(12), afad221.* *https://doi.org/10.1093/ageing/afad221*

Theou, O., van der Valk, A. M., Godin, J., Andrew, M. K., McElhaney, J. E., McNeil, S. A., & Rockwood, K. (2020). Exploring Clinically Meaningful Changes for the Frailty Index in a Longitudinal Cohort of Hospitalized Older Patients. *The Journals of Gerontology Series A: Biological Sciences and Medical Sciences,* 75(10), 1928–1934. https://doi.org/10.1093/gerona/glaa084

Supplementary Table 3.3. Estimated coefficients, 95% confidence intervals, and correlations from pooled multivariate growth curve model, *for Western, Southern, and Northern European regions*, using data from the Survey of Health, Aging, and Retirement in Europe, 2004-2022

**Fixed-effects parameters**				
		Estimate	*95% CI*	
Wealth	Intercept	7.71	5.47 - 9.95	
	Age	1.46	1.36 - 1.56	
	Age^2	0.05	0.04 - 0.06	
Health	Intercept	10.09	10.04 - 10.14	
	Age	-0.07	-0.07 - -0.06	
	Age^2	-0.00	-0.00 - -0.00	
**Random-effects parameters**				
Level 2: individual		*Estimate*	*95% CI*	*Correlation*
Variance	Wealth intercept	1043.83	1015.67 - 1072.00	
	Wealth slope	7.04	6.26 - 7.81	
	Health intercept	1.16	1.14 - 1.18	
	Health slope	0.01	0.01 - 0.01	
Covariance	Intercept-slope in wealth ($\sigma_{u1,u0}$)	57.92	54.45 - 61.39	0.68
	Intercept-slope in health ($\sigma_{u3,u2}$)	0.05	0.05 - 0.06	0.57
	Intercepts across domains ($\sigma_{u2,u0}$)	3.38	2.86 - 3.89	0.10
	Intercept wealth – slope health ($\sigma_{u3,u0}$)	0.33	0.26 - 0.39	0.11
	Intercept health – slope wealth ($\sigma_{u2,u1}$)	0.13	0.04 - 0.22	0.05
	Slopes across domains ($\sigma_{u3,u1}$)	0.02	0.01 - 0.03	0.07
Level 1: observation		*Correlation*	*Estimate*	*95% CI*
Variance	Wealth residuals	1303.53	1269.62 - 1337.44	
	Health residuals	0.24	0.24 - 0.24	
Covariance	Residuals across domains	-0.11	-0.28 - 0.06	-0.01

Note. The control variables woman, married or partnered, no children, co-residence with children, rural, born in survey country, working, household equivalized income, education, country, and wave are included in the model. Age centered at 57.4 years. Countries included are: Austria, Belgium, Cyprus, Denmark, Finland, France, Germany, Greece, Israel, Italy, Luxembourg, Malta, the Netherlands, Portugal, Spain, Sweden, and Switzerland.

Supplementary Table 3.4. Estimated coefficients, 95% confidence intervals, and correlations from pooled multivariate growth curve model of *total wealth* and health, using data from the Survey of Health, Aging, and Retirement in Europe, 2004-2022

**Fixed-effects parameters**				
		Estimate	*95% CI*	
Wealth	Intercept	84.24	69.12 - 99.36	
	Age	4.68	4.17 - 5.19	
	Age^2	0.07	0.01 - 0.14	
Health	Intercept	10.12	10.06 - 10.18	
	Age	-0.07	-0.07 - -0.07	
	Age^2	-0.00	-0.00 - -0.00	
**Random-effects parameters**				
Level 2: individual		*Estimate*	*95% CI*	*Correlation*
Variance	Wealth intercept	32392.63	31482.98 - 33302.29	
	Wealth slope	173.17	148.49 - 197.84	
	Health intercept	1.16	1.15 - 1.18	
	Health slope	0.01	0.01 - 0.01	
Covariance	Intercept-slope in wealth ($\sigma_{u1,u0}$)	119.79	7.69 - 231.89	0.05
	Intercept-slope in health ($\sigma_{u3,u2}$)	0.05	0.05 - 0.06	0.57
	Intercepts across domains ($\sigma_{u2,u0}$)	21.83	19.34 - 24.32	0.11
	Intercept wealth – slope health ($\sigma_{u3,u0}$)	1.62	1.31 - 1.93	0.10
	Intercept health – slope wealth ($\sigma_{u2,u1}$)	0.46	0.00 - 0.91	0.03
	Slopes across domains ($\sigma_{u3,u1}$)	0.03	-0.03 - 0.09	0.03
Level 1: observation		*Estimate*	*95% CI*	*Correlation*
Variance	Wealth residuals	43659.86	42082.48 - 45237.25	
	Health residuals	0.25	0.25 - 0.26	
Covariance	Residuals across domains	0.34	-0.71 - 1.38	0.00

Note. The control variables woman, married or partnered, no children, co-residence with children, rural, born in survey country, working, household equivalized income, education, country, and wave are included in the model. Age is centered at 57.4 years.

Supplementary Table 3.5. Estimated coefficients, 95% confidence intervals, and correlations from pooled multivariate growth curve model of *liquid wealth* and health, *with* age^2 random effects, using data from the Survey of Health, Aging, and Retirement in Europe, 2004-2022

**Fixed-effects parameters**				
		Estimate	*95% CI*	
Wealth	Intercept	-2.36	-4.51 - -0.20	
	Age	1.12	1.04 - 1.20	
	Age^2	0.04	0.03 - 0.04	
Health	Intercept	10.13	10.07 - 10.18	
	Age	-0.07	-0.07 - -0.07	
	Age^2	-0.00	-0.00 - -0.00	
**Random-effects parameters**				
Level 2: individual		*Estimate*	*95% CI*	*Correlation*
Variance	Wealth intercept	693.29	672.24 - 714.35	
	Wealth slope	8.93	8.17 - 9.68	
	Wealth slope^2	0.05	0.03 - 0.06	
	Health intercept	1.25	1.23 - 1.27	
	Health slope	0.01	0.01 - 0.01	
	Health slope^2	0.00	0.00 - 0.00	
Covariance	Intercept-slope in wealth ($\sigma_{u1,u0}$)	18.35	15.60 - 21.10	0.23
	Intercept-slope in health ($\sigma_{u3,u2}$)	0.04	0.04 - 0.04	0.39
	Intercepts across domains ($\sigma_{u2,u0}$)	2.66	2.21 - 3.10	0.09
	Intercept wealth – slope health ($\sigma_{u3,u0}$)	0.24	0.19 - 0.30	0.09
	Intercept health – slope wealth ($\sigma_{u2,u1}$)	0.08	-0.01 - 0.16	0.02
	Slopes across domains ($\sigma_{u3,u1}$)	0.01	0.00 - 0.02	0.05
	Intercept-slope^2 in wealth	-1.69	-2.23 - -1.14	
	Intercept-slope^2 in health	-0.00	-0.00 - -0.00	
	Intercept wealth – slope^2 health	0.00	-0.00 - 0.01	
	Intercept health – slope^2 wealth	0.00	-0.01 - 0.02	
	Slope wealth – slope^2 health	0.00	-0.00 - 0.00	
	Slope^2 wealth – slope health	-0.00	-0.00 - 0.00	
	Slope-slope^2 in wealth	0.51	0.44 - 0.59	
	Slope-slope^2 in health	0.00	0.00 - 0.00	
	Slope^2 across domains	-0.00	-0.00 - 0.00	
Level 1: observation		*Estimate*	*95% CI*	*Correlation*
Variance	Wealth residuals	1015.86	988.67 - 1043.06	
	Health residuals	0.23	0.23 - 0.23	
Covariance	Residuals across domains	-0.06	-0.20 - 0.09	0.00

Note. The control variables woman, married or partnered, no children, co-residence with children, rural, born in survey country, working, household equivalized income, education, country, and wave are included in the model. Age is centered at 57.4 years.

Supplementary Table 3.6. Estimated coefficients, 95% confidence intervals, and correlations from pooled multivariate growth curve model of liquid wealth and *age centered at 50 years*, using data from the Survey of Health, Aging, and Retirement in Europe, 2004-2022

**Fixed-effects parameters**				
		Estimate	*95% CI*	
Wealth	Intercept	-8.67	-10.83 - -6.51	
	Age (50 years)	0.57	0.43 - 0.72	
	Age^2 (50 years)	0.04	0.03 - 0.05	
Health	Intercept	10.59	10.53 - 10.65	
	Age (50 years)	-0.06	-0.06 - -0.05	
	Age^2 (50 years)	-0.00	-0.00 - -0.00	
**Random-effects parameters**				
Level 2: individual		*Estimate*	*95% CI*	*Correlation*
Variance	Wealth intercept	492.11	455.83 - 528.40	
	Wealth slope	5.86	5.31 - 6.42	
	Health intercept	0.79	0.77 - 0.81	
	Health slope	0.01	0.01 - 0.01	
Covariance	Intercept-slope in wealth ($\sigma_{u1,u0}$)	-11.23	-15.02 - -7.44	-0.21
	Intercept-slope in health ($\sigma_{u3,u2}$)	-0.00	-0.01 - -0.00	-0.06
	Intercepts across domains ($\sigma_{u2,u0}$)	1.29	0.74 - 1.84	0.07
	Intercept wealth – slope health ($\sigma_{u3,u0}$)	0.12	0.05 - 0.18	0.06
	Intercept health – slope wealth ($\sigma_{u2,u1}$)	-0.03	-0.10 - 0.04	-0.01
	Slopes across domains ($\sigma_{u3,u1}$)	0.02	0.01 - 0.03	0.07
Level 1: observation		*Estimate*	*95% CI*	*Correlation*
Variance	Wealth residuals	1062.09	1035.46 - 1088.72	
	Health residuals	0.25	0.25 - 0.26	
Covariance	Residuals across domains	-0.10	-0.24 - 0.04	-0.01

Note. The control variables woman, married or partnered, no children, co-residence with children, rural, born in survey country, working, household equivalized income, education, country, and wave are included in the model.

Supplementary Table 3.7. Estimated coefficients, 95% confidence intervals, and correlations from pooled multivariate growth curve model with *one partner randomly selected from each couple*, using data from the Survey of Health, Aging, and Retirement in Europe, 2004-2022

**Fixed-effects parameters**				
		Estimate	*95% CI*	
Wealth	Intercept	-2.29	-5.11 - 0.54	
	Age	1.16	1.05 - 1.26	
	Age^2	0.03	0.02 - 0.05	
Health	Intercept	10.11	10.04 - 10.19	
	Age	-0.07	-0.08 - -0.07	
	Age^2	-0.00	-0.00 - -0.00	
**Random-effects parameters**				
Level 2: individual		*Estimate*	*95% CI*	*Correlation*
Variance	Wealth intercept	737.27	710.39 - 764.15	
	Wealth slope	5.84	5.13 - 6.55	
	Health intercept	1.20	1.18 - 1.22	
	Health slope	0.01	0.01 - 0.01	
Covariance	Intercept-slope in wealth ($\sigma_{u1,u0}$)	33.32	30.16 - 36.47	0.51
	Intercept-slope in health ($\sigma_{u3,u2}$)	0.06	0.05 - 0.06	0.56
	Intercepts across domains ($\sigma_{u2,u0}$)	3.12	2.59 - 3.65	0.10
	Intercept wealth – slope health ($\sigma_{u3,u0}$)	0.28	0.21 - 0.34	0.11
	Intercept health – slope wealth ($\sigma_{u2,u1}$)	0.08	-0.02 - 0.18	0.03
	Slopes across domains ($\sigma_{u3,u1}$)	0.01	0.00 - 0.03	0.06
Level 1: observation		*Estimate*	*95% CI*	*Correlation*
Variance	Wealth residuals	1185.67	1160.10 - 1211.23	
	Health residuals	0.26	0.25 - 0.26	
Covariance	Residuals across domains	-0.08	-0.27 - 0.11	0.00

Note. The control variables woman, married or partnered, no children, co-residence with children, rural, born in survey country, working, household equivalized income, education, country, and wave are included in the model. Age centered at 57.5 years.

Supplementary Table 3.8. Estimated coefficients, 95% confidence intervals, and correlations from pooled multivariate growth curve model, *controlling for occupation type among participants who reported working in their entry wave*, using data from the Survey of Health, Aging, and Retirement in Europe, 2004-2022

**Fixed-effects parameters**				
		Estimate	*95% CI*	
Wealth	Intercept	-5.25	-41.88 - 31.38	
	Age	1.12	1.00 - 1.24	
	Age^2	0.04	0.03 - 0.06	
Health	Intercept	10.18	9.71 - 10.64	
	Age	-0.06	-0.06 - -0.06	
	Age^2	-0.00	-0.00 - -0.00	
**Random-effects parameters**				
Level 2: individual		*Estimate*	*95% CI*	*Correlation*
Variance	Wealth intercept	732.44	698.45 - 766.43	
	Wealth slope	6.54	5.37 - 7.71	
	Health intercept	0.91	0.89 - 0.92	
	Health slope	0.01	0.01 - 0.01	
Covariance	Intercept-slope in wealth ($\sigma_{u1,u0}$)	38.55	34.41 - 42.70	0.56
	Intercept-slope in health ($\sigma_{u3,u2}$)	0.04	0.04 - 0.05	0.58
	Intercepts across domains ($\sigma_{u2,u0}$)	1.84	1.36 - 2.32	0.07
	Intercept wealth – slope health ($\sigma_{u3,u0}$)	0.16	0.10 - 0.23	0.08
	Intercept health – slope wealth ($\sigma_{u2,u1}$)	0.03	-0.06 - 0.13	0.01
	Slopes across domains ($\sigma_{u3,u1}$)	0.01	-0.00 - 0.02	0.04
Level 1: observation		*Estimate*	*95% CI*	*Correlation*
Variance	Wealth residuals	1207.21	1164.32 - 1250.09	
	Health residuals	0.20	0.19 - 0.20	
Covariance	Residuals across domains	-0.04	-0.26 - 0.17	0.00

Note. The control variables woman, married or partnered, no children, co-residence with children, rural, born in survey country, working, household equivalized income, education, country, and wave are included in the model. Age centered at 56.9 years.

Supplementary Table 3.9. Estimated coefficients, 95% confidence intervals, and correlations from pooled multivariate growth curve model, *controlling for smoking and physical activity*, using data from the Survey of Health, Aging, and Retirement in Europe, 2004-2022

**Fixed-effects parameters**				
		Estimate	*95% CI*	
Wealth	Intercept	3.15	0.72 - 5.57	
	Age	1.12	1.04 - 1.20	
	Age^2	0.04	0.03 - 0.05	
Health	Intercept	10.26	10.20 - 10.33	
	Age	-0.07	-0.07 - -0.07	
	Age^2	-0.00	-0.00 - -0.00	
**Random-effects parameters**				
Level 2: individual		*Estimate*	*95% CI*	*Correlation*
Variance	Wealth intercept	646.35	628.70 - 663.99	
	Wealth slope	5.50	4.91 - 6.10	
	Health intercept	1.14	1.12 - 1.15	
	Health slope	0.01	0.01 - 0.01	
Covariance	Intercept-slope in wealth ($\sigma_{u1,u0}$)	34.14	31.57 - 36.71	0.57
	Intercept-slope in health ($\sigma_{u3,u2}$)	0.05	0.05 - 0.06	0.58
	Intercepts across domains ($\sigma_{u2,u0}$)	2.54	2.16 - 2.92	0.09
	Intercept wealth – slope health ($\sigma_{u3,u0}$)	0.23	0.18 - 0.28	0.10
	Intercept health – slope wealth ($\sigma_{u2,u1}$)	0.09	0.02 - 0.17	0.04
	Slopes across domains ($\sigma_{u3,u1}$)	0.02	0.01 - 0.03	0.08
Level 1: observation		*Estimate*	*95% CI*	*Correlation*
Variance	Wealth residuals	1093.39	1061.70 - 1125.08	
	Health residuals	0.25	0.25 - 0.26	
Covariance	Residuals across domains	-0.09	-0.24 - 0.07	-0.01

Note. The control variables woman, married or partnered, no children, co-residence with children, rural, born in survey country, working, household equivalized income, education, country, and wave are included in the model. Age centered at 57.4 years.

Supplementary Table 3.10. Estimated coefficients, 95% confidence intervals, and correlations from pooled multivariate growth curve model, *stratified by working status*, using data from the Survey of Health, Aging, and Retirement in Europe, 2004-2022

		Working			Not working		
**Fixed-effects parameters**		*Estimate*	*95% CI*		*Estimate*	*95% CI*	
Wealth	Intercept	-1.72	-4.67 - 1.23		-1.67	-4.59 - 1.24	
	Age	1.26	1.15 - 1.37		1.05	0.95 - 1.15	
	Age^2	0.05	0.04 - 0.07		0.04	0.02 - 0.05	
Health	Intercept	10.28	10.21 - 10.35		10.19	10.09 - 10.29	
	Age	-0.07	-0.07 - -0.06		-0.06	-0.07 - -0.06	
	Age^2	-0.00	-0.00 - -0.00		-0.00	-0.00 - -0.00	
**Random-effects parameters**							
Level 2: individual		*Estimate*	*95% CI*	*Correlation*	*Estimate*	*95% CI*	*Correlation*
Variance	Wealth intercept	753.36	723.74 - 782.97		478.00	442.39 - 513.62	
	Wealth slope	7.05	6.10 - 8.01		3.16	2.57 - 3.76	
	Health intercept	1.02	1.00 - 1.04		1.38	1.35 - 1.41	
	Health slope	0.01	0.01 - 0.01		0.01	0.01 - 0.01	
Covariance	Intercept-slope in wealth ($\sigma_{u1,u0}$)	42.86	39.04 - 46.67	0.59	35.70	32.56 - 38.84	0.92
	Intercept-slope in health ($\sigma_{u3,u2}$)	0.05	0.05 - 0.05	0.61	0.04	0.04 - 0.05	0.40
	Intercepts across domains ($\sigma_{u2,u0}$)	2.34	1.88 - 2.81	0.08	3.24	2.67 - 3.81	0.13
	Intercept wealth – slope health ($\sigma_{u3,u0}$)	0.20	0.14 - 0.26	0.09	0.23	0.15 - 0.31	0.11
	Intercept health – slope wealth ($\sigma_{u2,u1}$)	0.05	-0.04 - 0.14	0.02	0.07	-0.04 - 0.18	0.03
	Slopes across domains ($\sigma_{u3,u1}$)	0.01	0.00 - 0.02	0.05	0.02	0.01 - 0.03	0.13
Level 1: observation		*Estimate*	*95% CI*	*Correlation*	*Estimate*	*95% CI*	*Correlation*
Variance	Wealth residuals	1198.01	1155.51 - 1240.51		816.22	773.33 - 859.11	
	Health residuals	0.20	0.20 - 0.21		0.33	0.32 - 0.33	
Covariance	Residuals across domains	-0.07	-0.25 - 0.12	0.00	-0.20	-0.49 - 0.08	-0.01

Note. The control variables woman, married or partnered, no children, co-residence with children, rural, born in survey country, working, household equivalized income, education, country, and wave are included in the model. Age centered at 57.4 years.
